# What about the treatment of asymptomatic forms of congenital malaria: case report and review of the literature

**DOI:** 10.11604/pamj.2020.35.116.16628

**Published:** 2020-04-14

**Authors:** Salahiddine Saghir, Mounir Moukit, Jaouad Kouach, Naoufal Assoufi, Rachid Abilkassem, Aomar Agadr

**Affiliations:** 1 Pediatric Department, Military Hospital Mohammed V, Rabat, Morocco; 2 Obstetrics and Gynecology Department, Military Hospital Mohammed V, Rabat, Morocco; 3 Department of Internal Medicine, Military Hospital Mohammed V, Rabat, Morocco

**Keywords:** Congenital malaria, *Plasmodium falciparum*, parasitemia, malaria infestation, chloroquine

## Abstract

We report in this manuscript a case of newborn baby with asymptomatic form of congenital malaria; the screening of the peripheral blood smear of the baby after a positive result in the mother allowed the diagnosis. The authors were permitted through this case to discuss the therapeutic possibility in these cases.

## Introduction

In congenital malaria, infants are infected by the passage of parasites from mother to child before or during birth, it is a fatal condition that occurs at low rates in endemic countries [1]. Malaria is defined as an important public health problem in sub-Saharan Africa endemic countries to *Plasmodium falciparum*, recent data suggest that this is a more frequent situation than expected [2]. In these areas, even if the risk of contamination from the mother is high, newborns are protected against congenital malaria by maternal antibodies.

We report a case of asymptomatic congenital malaria; to our knowledge the articles available in the literature talk about symptomatic cases of the disease. On the other hand, the authors through this case discuss the therapeutic possibilities of asymptomatic congenital malaria. Congenital malaria is the presence of malarial parasites in the peripheral blood smear of the newborn from the first day until the seventh day of life [3]. It is due to transplacental transfer of parasites which infect the infant in utero or during delivery. In endemic regions congenital malaria is principally caused by *Plasmodium falciparum* [4]. In these countries mortality is over 86% in children under 5 years of age [5, 6]. The most common clinical symptoms are fever, anaemia, and splenomegaly in 80% of cases [7, 8].

## Patient and observation

A 32 years old woman was admitted during labor, to the Department of Gynecology of the Moroccan Field hospital deployed in South Sudan. The patient was gravida 3, para 2, abortus 1 (2 living children) at the 39^th^ week of gestation, prenatal screening tests were not performed. The clinical examination showed normal fundal height, with perception of uterine contractions and normal fetal heart activity, the cervix was effaced and dilated to 4cm. Trans-abdominal ultrasound was performed; the placenta was localized in the posterior side of the fundus; the amniotic fluid and the fetal growth were normal. A healthy male infant weighing 2600g was successfully delivered via spontaneous vaginal delivery, the apgar score was 8/9/10 with no blood loss during delivery, and no congenital abnormality was detected. 24 hours after delivery, the mother presented acute fever associated to chills and excessive sweating; she had anemia with hemoglobin 8.2g/dl, hematocrit 25.8%, MCV 86.1fl, MCH 28.5pg, MCHC 31.5g/dl, total leucocyte count 5.5×10^3^/ul and platelet count was low 56,000/ul. C-reactive protein (CRP) was normal (5,2mg/l), with normal range of serum electrolytes. Blood and urine cultures were sterile and peripheral blood smear showed malarial parasites of *Plasmodium falciparum* parasitemia at 4%.

The mother reported after evaluation a possible malaria infection in the 32^th^ week of pregnancy, managed with unspecific treatment. The newborn baby was healthy and vigorous, exclusively breastfed, the clinical examination was normal; biological analysis showed hemoglobin 16.3g/dl, total leucocyte count 7.5×10^3^/ul and platelet count 72,000/ul. C-reactive protein (CRP) was normal (< 5mg/l), with normal range of serum electrolytes. Peripheral blood smear was negative for malaria parasites; unfortunately, the other methods of diagnosis (Detection of PfHRP2 antigen, DNA detection and quantification) were not available for further investigations, the newborn was placed under close observation and a second examination of the peripheral blood, was done within 3 days; a thick and thin blood film revealed *P. falciparum trophozoites*, with a parasitaemia of 1,5%. The mother was treated according to local guidelines with arthemeter + lumefantrine and the infant was treated by oral chloroquine 10mg/kg for two days and 5mg/kg in the third day. Four days after treatment haemoparasites were cleared, with normal follow-up.

## Discussion

Congenital malaria was described the first time in 1876; it is defined by the presence of asexual forms of malaria parasites in peripheral blood within the first week of life [9]. The occurrence of congenital malaria remains rare. The asymptomatic form is the most frequently reported [10], only 7 to 10% of newborns develop the disease form [11, 12]. The physiopathology still misunderstood, possible theories include transfusion of the fetal circulation by maternal blood during pregnancy, direct penetration in the chorionic villi due to premature separation of the placenta [13]. Clinical signs in order of frequency are anemia, fever, hepatosplenomegaly, hypotonia (poor feeding, lethargy), irritability and jaundice [14, 15]. Severe thrombocytopenia is also frequently reported [16]. The diagnosis is simple, based on microscopic examination of peripheric blood films, other diagnostic methods are possible if the blood film examination is negative: RDT (rapid diagnostic test) or PCR (polymerase chain reaction) [17]. In our case, the first thick blood smear examination was negative and could be explained by several factors such as fetal hemoglobin composition [18], protection by maternal antibodies specific to malaria after passive transplacental transfer, exclusive breastfeeding [19] and by the reduction of red blood cells in the circulation due to their sequestration [20]. The second examination was positive at the age of 5 days [21].

The genomic amplification and DNA quantification increases the prevalence of diagnosis but they were not possible in our conditions. There are no clearly guidelines established to treat congenital malaria. Primaquine is not indicated because the infection is acquired congenitally (it doesn't include the persistent hepatic phase); chloroquine has been the drug of choice in case of congenital falciparum malaria [22]. However, a high incidence of chloroquine resistance has been reported by a recent study; in Nigeria 89.1% of patients were resistant to oral chloroquine, it was replaced by the association of sulfadoxine-pyrimethamine with good result [23]. Quinine plus clindamycin was also used in another study and have been reported as an effective treatment [24]. The efficacy of artesunate over quinine have been demonstrated [25]. The authors noted that it can be used as drug of first choice to treat congenital malaria. Preventive therapy using the association sulfadoxine-pyrimethamine to pregnant women during the second and third trimester, according to the world health was beneficial [26]. For patients who develop the symptomatic form of congenital malaria, the morbidity and the mortality associated is very high; although, in different studies, the majority of the infected neonates were asymptomatic during the observation period.

However, in various studies, the cases of congenital falciparum malaria, whether symptomatic or not, should be treated [11, 27, 28], but unfortunately none of the patients was followed until the 15th week corresponding to the necessary time for the elimination of the passive immunity transmitted by the mother [29], therefore the clinical evaluation of the asymptomatic patients was incomplete and they didn't receive any treatment. In our case, the patient received his treatment because the access to hospitals and healthcare facilities in such an emerging country was very difficult which impeded regular clinical monitoring and was impossible for him.

## Conclusion

The repetition of thick drop examination has helped us to assert the diagnosis of congenital malaria in asymptomatic and healthy patient. When close clinical monitoring of patients over a 15-week period is feasible, antimalarial treatment should not be initiated unless a clinical signs of infection occurs and after diagnostic confirmation.

## Competing interests

The authors declare no competing interests.

## References

[1] Sule-Odu AO, Ogunledun A, Olatunji AO (2002). Impact of asymptomatic maternal malaria parasitaemia at parturition on perinatal outcome. Journal of obstetrics and gynaecology.

[2] Menendez C, Mayor A (2007). Congenital malaria: the least known consequence of malaria in pregnancy. Semin Fetal Neonatal Med.

[3] Arvin AM, Maldonado YA, Remington JS, Klein JO (1995). Protozoan and Helminth Infections. Infectious diseases of the fetus and newborn infant.

[4] Ibhanesebhor SE (2005). Clinical characteristics of neonatal malaria. J Trop Pediatr.

[5] World Health Organization (2012). World Malaria Report 2012.

[6] Eisele TP, Larsen DA, Walker N, Cibulskis RE, Yukich JO (2012). Estimates of child deaths prevented from malaria prevention scale-up in Africa. 2001-2010. Malar J.

[7] Krause PJ, Behrman RE, Keligman R, Jenson HB (2004). Malaria (Plasmodium). Nelson Textbook of Pediatrics.

[8] Subramanian D, Moise KJ, White AC (1992). Imported malaria in pregnancy: report of four cases and review of management. Clin Infect Dis.

[9] Menendez C, Mayor A (2007). Congenital malaria: the least known consequence of malaria in pregnancy. Semin Fetal Neonatal Med.

[10] Falade C, Mokuolu O, Okafor H (2007). Epidemiology of congenital malaria in Nigeria: a multi-centre study. Trop Med Int Health.

[11] Obiajunwa PO, Owa JA, Adeodu OO (2005). Prevalence of congenital malaria in Ile-Ife, Nigeria. J Trop Pediatr.

[12] McGregor A (1984). Epidemiology, malaria and pregnancy. Am J Trop Med Hyg.

[13] De Silva DH, Mendis KN, Premaratne UN, Jayatilleke SM, Soyza PE (1982). Congenital malaria due to plasmodium vivax: a case report from Sri Lanka. Trans R Soc Trop Med Hyg.

[14] Voittier G, Arsac M, Farnoux C, Mariani-Kurdjian P, Baud O, Aujard Y (2008). Congenital malaria in neonates: two case report and review of literature. Acta Paediatr.

[15] Fischer PR (2003). Malaria and newborns. Journal of Tropical Pediatrics.

[16] Baspinar O, Bayraktaroglu Z, Karsligil T, Bayram A, Coscun Y (2006). A rare case of anemia and thrombocytopenia in a newborn: congenital malaria. Turkish Journal of Pediatrics.

[17] Wilson ML (2012). Malaria rapid diagnostic tests. Clinical Infectious Diseases.

[18] Miller IJ, Telford SR (1996). Placental malaria. N Eng J Med.

[19] Amaratunga C, Lopera-Mesa TM, Brittain NJ, Cholera R, Arie T, Fujioka H, Keefer JR, Fairhurts RM (2011). A role for fetal hemoglobin and maternal immune IgG in infant resistance to Plasmodium falciparum malaria. PLoS One.

[20] Udeniya IJ, Schmidt JA, Aikawa M (1981). Falciparum malaria-infected erythrocytes specifically bind to cultured human endothelial cells. Science.

[21] Piñeros-Jiménez JG, Alvarez G, Tobon A (2011). Congenital malaria in Urabá, Colombia. Malaria Journal.

[22] Del Punta V, Gulletta M, Matteelli A, Spinoni V, Regazzoli A, Castelli F (2010). Congenital plasmodium vivax malaria mimicking neonatal sepsis: a case report. Malaria J.

[23] Orogade AA, Falade CO, Okafor HU, Mokuolu OA, Mamman AI (2008). Clinical and laboratory features of congenital malaria in Nigeria. Journal of Pediatric Infectious Diseases.

[24] Harrington WE, Duffy PE (2008). Congenital malaria: rare but potentially fatal. Pediatric Health.

[25] Patel AB, Belsare HS (2002). Resistant malaria in a neonate. Indian Pediatrics.

[26] WHO (2012). Evidence review group: intermittent preventive treatment in pregnancy (IPTp) with sulfadoxine-pyrimethamine (SP).

[27] Larkin GL, Thuma PE (1991). Congenital malaria in a hyperendemic area. Am J Trop Med Hyg.

[28] Dao N Ginette L, Zohoncon TM (2014). Mother-to-children plasmodium falciparum asymptomatic malaria transmission at Saint Camille Medical Centre in Ouagadougou, Burkina Faso. Malaria research and treatment.

[29] Quinn TC, Jacobs RF, Mertz GJ, Hook EW, Locklsey RM (1982). Congenital malaria a report of four cases and a review. J Pediatr.

